# Evidence against apoptosis as a major mechanism for reproductive cell death following treatment of cell lines with anti-cancer drugs

**DOI:** 10.1054/bjoc.2000.1538

**Published:** 2001-01-01

**Authors:** I F Tannock, C Lee

**Affiliations:** Dept of Medical Oncology and Haematology, and the Division of Experimental Therapy, Ontario Cancer Institute and Princess Margaret Hospital, University of Toronto, Toronto, Canada

**Keywords:** apoptosis, anti-cancer drugs, clonogenic survival

## Abstract

An increase in apoptotic cells may be observed after treatment with chemotherapy, and many authors have assumed that anti-cancer drugs kill cells by inducing apoptosis. The most relevant endpoint of cell death following treatment of tumour cells is loss of reproductive ability as measured by a colony-forming assay, since cells with limited reproductive potential cannot regenerate a tumour. We have therefore investigated the relationship between apoptosis and reproductive cell death following in vitro treatment of mammalian cell lines with anti-cancer drugs. Markers of apoptosis (DNA ladders, TUNEL assay) were evaluated at various times after treatment of Chinese Hamster Ovary (CHO) cells, human bladder cancer MGH-U1 cells, and a murine T-lymphocytic cell line (CTLL-2) with several anti-cancer drugs. These markers were found infrequently, despite the use of doses that cause loss of colony-forming ability, except in CTLL-2 cells. We also transfected and expressed the human pro-apoptotic gene *bax* and the anti-apoptotic gene *bcl-2* in MGH-U1 cells and compared cell survival after drug treatment with that of control cells transfected with the vector alone. Expression of these genes had at most small effects to influence cell survival. We conclude that apoptotic mechanisms had at most a minor role in leading to reproductive death of MGH-U1 and CHO cells after chemotherapy. When apoptosis is observed following treatment with anti-cancer drugs it may be a secondary event which occurs in lethally-damaged cells, leading to their lysis, rather than a primary event that leads to loss of reproductive integrity. © 2001 Cancer Research Campaign http://www.bjcancer.com


British Journal of Cancer (2001) **84**(1), 100–105
© 2001 Cancer Research Campaign
doi: 10.1054/ bjoc.2000.1538, available online at http://www.idealibrary.com on IDEAL®

http://www.bjcancer.com

### Evidence against apoptosis as a major mechanism for reproductive cell death following treatment of cell lines with anti-cancer drugs

**IF Tannock and C Lee**

Dept of Medical Oncology and Haematology, and the Division of Experimental Therapy, Ontario Cancer Institute and Princess Margaret Hospital, University of Toronto, Toronto, Canada

**Summary** An increase in apoptotic cells may be observed after treatment with chemotherapy, and many authors have assumed that anti-cancer drugs kill cells by inducing apoptosis. The most relevant endpoint of cell death following treatment of tumour cells is loss of reproductive ability as measured by a colony-forming assay, since cells with limited reproductive potential cannot regenerate a tumour. We have therefore investigated the relationship between apoptosis and reproductive cell death following in vitro treatment of mammalian cell lines with anti-cancer drugs. Markers of apoptosis (DNA ladders, TUNEL assay) were evaluated at various times after treatment of Chinese Hamster Ovary (CHO) cells, human bladder cancer MGH-U1 cells, and a murine T-lymphocytic cell line (CTLL-2) with several anti-cancer drugs. These markers were found infrequently, despite the use of doses that cause loss of colony-forming ability, except in CTLL-2 cells. We also transfected and expressed the human pro-apoptotic gene *bax* and the anti-apoptotic gene *bcl-2* in MGH-U1 cells and compared cell survival after drug treatment with that of control cells transfected with the vector alone. Expression of these genes had at most small effects to influence cell survival. We conclude that apoptotic mechanisms had at most a minor role in leading to reproductive death of MGH-U1 and CHO cells after chemotherapy. When apoptosis is observed following treatment with anti-cancer drugs it may be a secondary event which occurs in lethally-damaged cells, leading to their lysis, rather than a primary event that leads to loss of reproductive integrity. © 2001 Cancer Research Campaign http://www.bjcancer.com

**Keywords**: apoptosis; anti-cancer drugs; clonogenic survival

There is a very large literature describing the appearance of various markers of apoptosis following treatment of cells with anti-cancer drugs. In the majority of these studies there is either an explicit statement or an implicit assumption that chemotherapy has led to cell death by initiating an apoptotic pathway. However, as pointed out by Smets (1994) and others (Brown and Wouters, 1999), activation of the apoptotic pathway might occur either (A) as a primary event induced by therapy, or (B) as a secondary event following lethal damage to the cell (Fig. 1). This distinction is not trivial, because if apoptosis occurs as a primary event following chemotherapy (or after treatment with radiation or other agents), then the effectiveness of treatment will depend on the cell's ability to activate the apoptotic pathway, and hence on the expression of genes that initiate, regulate or mediate the pathway. The effectiveness of treatment could, in principle, be modified through up-or down-regulation of the apoptotic pathway, and mutations leading to defective apoptosis would have the potential for causing drug resistance. In contrast, if apoptosis were a secondary event which occurred in cells that had sustained lethal damage (e.g. to DNA) and merely controlled the rate of lysis of cells that had already lost their reproductive potential, manipulation of the pathway would have no influence on the long-term effects of treatment.

Most studies that have investigated the appearance of apoptotic changes following treatment of malignant cells with anti-cancer drugs have used either an 'apoptotic index' as the endpoint of cell

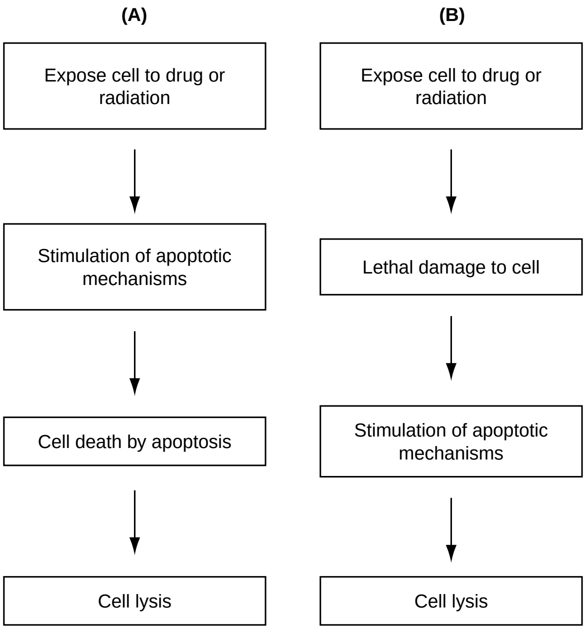

**Figure 1** Pathways that might lead to apoptotic cells following cancer treatment. (**A**) Apoptosis is induced by radiation or drugs and leads to cell death. The process might then be modified to influence sensitivity to treatment. (**B**) Radiation or drugs cause lethal damage to the cell, which in turn induces apoptosis as a mechanism of cell lysis. Modification of apoptosis will then influence the timing of cell lysis but not the proportion of surviving cells

*Received 24 March 2000*
*Revised 14 September 2000*
*Accepted 20 September 2000*

*Correspondence to:* IF Tannock death (e.g. the proportion of cells that are positive in an assay for apoptosis, such as TUNEL assay), or a simple measure of 'cell viability' such as exclusion of trypan blue or another dye. However, the measure of cell death that is most relevant for a population of tumour cells is reproductive cell death as measured by a colony-forming assay: a cell that cannot produce a colony of progeny under optimal conditions is unlikely to be able to reproduce the tumour. There is a substantial, although older, literature (e.g. Roper and Drewinko, 1976) that has shown very poor agreement between the assessment of cell death by loss of clonogenic capacity, and morphological markers or the inability to exclude dyes. In general, cells that have sustained a lethal event may retain their morphological appearance and exclude dyes for some period after they have lost their reproductive potential. Moreover, successful treatment with an anti-cancer drug implies reduction in survival on a logarithmic scale (i.e. doses tolerated in vivo should reduce survival to the range of $10^{-2}$ to $10^{-3}$ or lower) whereas properties such as apoptotic index, morphological change or loss of dye exclusion are inherently limited to a linear scale. Changes in 'viability' assessed by these methods are difficult or impossible to measure below a level of about 5–10%.

A few studies have examined the relationship between apoptotic markers and clonogenic cell survival following treatment of cells with anti-cancer drugs or with radiation, and have come to conflicting conclusions: changes in expression of genes that regulate apoptosis, such as *bcl-2* or *bax* has led to changes in clonogenic survival after treatment with radiation or drugs in some studies (Hu et al, 1995; Sakakura et al, 1996) but have had no effect on clonogenic survival in others (Aldridge et al, 1995; Yin and Schimke 1995; Lock and Strabinskiene, 1996; Kyprianou et al, 1997; Beale et al, 1998). It is possible that publication bias may have limited the appearance of other studies that failed to show a correlation. In the present study we have evaluated markers of apoptosis following treatment with three types of cell with several anti-cancer drugs that are in common use. The cells selected for this study were (i) MGH-U1 human bladder cancer cells (also known as T24) which are known to contain wild type p53 (Chang and Lai, 2000); (ii) CHO Chinese hamster ovary cells known to contain mutant p53 (Hu et al, 1999); (iii) CTLL-2 murine T-cell leukaemia cell line, which is known to express apoptotic markers following a variety of stimuli, and which was used as a positive control to rule out technical problems in assays of apoptosis. We have used doses and exposure times that led to loss of reproductive integrity in a substantial proportion of the cells (>90% or at least a 10-fold reduction in survival). In addition we have sought evidence for a cause and effect relationship between apoptosis and loss of reproductive integrity by transfecting and expressing the genes *bcl-2* or *bax* in human cells and studying their clonogenic survival following treatment with doxorubicin and etoposide, two drugs which have been widely reported to cause cell death by apoptotic mechanisms. Our results suggest that apoptosis is not a major mechanism leading to cell killing by anti-cancer drugs for MGH-U1 and CHO cells.

### MATERIALS AND METHODS

### Cell lines

Human bladder cancer MGH-U1 cells were obtained originally from Dr G Prout (Massachusetts General Hospital, Boston, MA, USA), Chinese Hamster Ovary (CHO) cells were obtained originally from Dr LH Thompson (Lawrence Livermore Laboratories, CA, USA). These cell lines were maintained as monolayers in α- minimal essential medium (α-MEM) supplemented with 10% fetal calf serum (FCS), and were re-established from frozen stock at about 6-month intervals. The murine T-cell leukaemia cell line (CTLL-2) was obtained from the American Tissue Culture Collection (TIB214); this cell line was maintained in α-MEM +10% FCS supplemented with 50 μM interleukin 2 (IL2). Cell lines were checked routinely for the presence of mycoplasma, using the Hoechst 33258 fluorescent detection method, and were found to be negative.

### Anti-cancer drugs

We evaluated the effects of the following anti-cancer drugs on markers of apoptosis and on cell survival: cisplatin (Faulding, Vaudreuil, Quebec), cytosine arabinoside (Upjohn, Don Mills, ON), doxorubicin (Pharmacia, Mississauga, ON), etoposide (Novopharm, Toronto, ON) and gemcitabine (Eli Lilly, Indianapolis, Indiana). The drugs were prepared from our standard pharmacy formulations as used for patients, and then further diluted in phosphate buffered saline (PBS).

### Transfection of *bcl-2* and *bax* genes

MGH-U1 cells were stably transfected with *neo/pcDNA3*, full-length human *bcl-2/pcDNA3* or human *bax/pcDNA3* by lipofectamine and selected in 1 mg/ml G418. For each cell type, individual drug-resistant colonies were analysed by Western blot analysis using either anti-human bcl-2 (Dako, Mississauga, ON, Canada) or anti-human bax (Santa Cruz Biologicals, CA, USA) antibodies.

### Detection of apoptosis

Two methods were used to detect apoptosis in cells at various times after drug treatment: (I) the presence of 'DNA ladders' in DNA gel electrophoresis and (II) the Terminal deoxynucleotidyl transferase-mediated dUTP Nick End-Labelling (TUNEL) assay. Two positive controls were used for each of these assays to ensure that failure to detect apoptotic markers was not due to technical reasons; these were overgrown CHO cells in monolayer culture (i.e. grown beyond the stage of confluence), and CTLL-2 cells following withdrawal of IL2. Experiments are only reported here if the results were repeatable, and if concurrent positive controls showed unequivocal evidence of apoptosis.

For DNA gel electrophoresis, DNA was isolated and analysed as described previously (Brezden et al, 1994). Briefly, cells were trypsinized, collected in tissue culture tubes and washed with PBS before resuspension in 200 μl of 5 M guanidine thiocyanate containing 0.1 M 2-mercaptoethanol. DNA was precipitated by addition of 150 μl of 7.5 M ammonium acetate followed by 600 μl of 100% ethanol and incubated at –20˚C for at least 1 hour. Samples were centrifuged at 14 000 rpm for 30 min at 4˚C. The DNA pellet was washed twice with 500 μl of 75% ethanol, air-dried and redissolved in 10 mM Tris/1 mM EDTA at pH 8.0. The DNA was fractionated by electrophoresis through a 2% agarose gel prestained with ethidium bromide.

### The TUNEL assay

The TUNEL assay was performed by using the APO-BRDU kit (Phoenix Flow Systems, CA, USA). Briefly, cells were fixed in

© 2001 Cancer Research Campaign

1% paraformaldehyde in PBS for 15 min. Cells were then washed and labelled with DNA labelling solution containing TdT reaction buffer, TdT enzyme and Br-dUTP for 1 hour at 37˚C. At the end of incubation, cells were rinsed and stained with fluorescein~PRB-1 antibody and propidium iodide/RNase A solution for 30 min in the dark at room temperature. Br-dUTP is added to the 3′-OH ends of DNA fragments in apoptotic cells which can be recognized by their fluorescence. The population of apoptotic and non-apoptotic cells were distinguished by setting appropriate gates following flow cytometry.

### Assessment of cell survival by clonogenic assay

The survival of CHO cells and MGH-U1/neo cells following treatment with various anti-cancer drugs was assessed by a colony-forming assay. The survival of MGH-U1 cells transfected with *bax* or *bcl-2* genes was also compared with that of parental cells transfected with the control vector (*neo*) following a 1 hour exposure to varying doses of doxorubicin or etoposide.

Exponentially growing cells were trypsinized and cells at a concentration of $10^5$ $ml^{-1}$ were exposed to drugs. At the end of the incubations, cells were washed 3 × with PBS, trypsinized and resuspended in α-MEM. The cells were counted, serially diluted and plated in triplicate. Resulting colonies were stained with methylene blue and counted about 10 days later. Cell survival was expressed as the ratio of the plating efficiency of treated cells to that of untreated cells, and was plotted (using a logarithmic scale) against drug concentration. All experiments were performed at least twice.

### RESULTS

### Expression of *bax* and *bcl-2*

A Western blot showing expression of bax (p21) and bcl-2 (p26) proteins in MGH-U1 cells is shown in Figure 2. The level of expression of these proteins is increased substantially compared to cells transfected with vector alone (designated MGH-U1/neo).

### Apoptosis following drug treatment

Representative results of experiments using DNA electrophoresis are shown in Figure 3; DNA ladders indicating the presence of apoptosis were found reproducibly in gels for CTLL-2 cells following withdrawal of IL2 from the growth medium, and for CHO cells that were overgrown. As summarized in Tables 1–3, DNA ladders were found at 24 h following treatment of CTLL-2 cells with cisplatin and gemcitabine, but not at 1 h to 24 h following treatment with cytosine arabinoside, doxorubicin or etoposide. DNA ladders were not seen after treatment of MGH-U1 or CHO cells (including those transfected with *bcl-2* or *bax*) with any of the 5 anti-cancer drugs at various concentrations and exposure times.

Representative distributions of cells in the TUNEL assay are shown in Figure 4, and results following treatment of the 3 cell lines with anti-cancer drugs are summarized in Tables 1–3. The only TUNEL assays which demonstrated a substantial number of apoptotic cells were for overgrown CHO cells and for CTLL-2 cells after IL2 withdrawal. In addition to the conditions described in Tables 1–3, markers of apoptosis were not observed when CHO cells were treated with a variety of anti-cancer drugs and observed for periods of time ranging up to 6 days (data not shown).

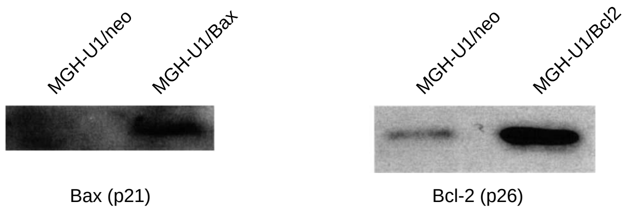

**Figure 2** Western blots indicating expression of bax and bcl-2 protein following transfection into MGH-U1 cells

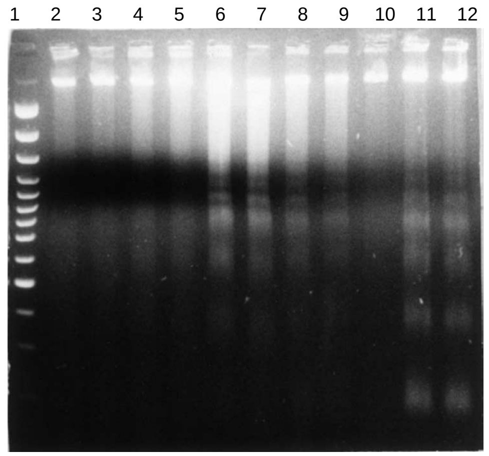

**Figure 3** DNA gel electrophoresis for CTLL-2 cells for the following conditions. (1) 100 bp DNA marker; (2 and 3) doxorubicin 0.9 and 1.8 μM respectively; (4 and 5) etoposide 20 and 40 μM respectively; (6 and 7) cisplatin 10 and 20 μM respectively; (8 and 9) gemcitabine 50 and 100 μM respectively; (10) control; (11) CHO overgrown cells and (12) withdrawal of IL2 from CTLL-2 cells. For duration of exposure and timing of assay, see Table 3. Ladders indicating presence of DNA breaks and apoptosis are evident only in lanes 6, 7, 8, 9, 11 and 12

### Assessment of cell survival

The surviving fractions of CHO and MGH-U1/neo cells following treatment with the various anti-cancer drugs are shown in Tables 1 and 2. Depending on the drug concentration and duration of treatment these drugs were able to cause substantial reduction in clonogenic survival (with the exception of treatment of MGH-U1 cells by cytosine arabinoside).

The pooled results of 3 experiments to evaluate the survival of MGH-U1 cells transfected with *bcl-2*, *bax* or the control vector (*neo*) following treatment with doxorubicin or etoposide are shown in Figure 5. There is no effect of *bcl-2* or *bax* expression on cell survival following treatment with etoposide; there is a small effect of *bcl-2* transfection to protect, and of *bax* transfection to enhance cell killing after treatment with doxorubicin.

### DISCUSSION

Understanding the role of apoptotic or other active mechanisms in cell death following treatment of cells with anti-cancer agents is of fundamental importance. If anti-cancer drugs or radiation cause damage to cells that can lead to either cell death or to repair of damage and viability, depending on the stimulation and activity of apoptotic pathways, then these pathways might be manipulated to

© 2001 Cancer Research Campaign

**Table 1** Summary of experiments to detect apoptotic markers and survival fraction (using a colony-forming assay) for MGH-U1/neo cells treated with a variety of anti-cancer drugs. Treatment of MGH-U1/bax and MGH-U1/bcl-2 cells under the same conditions also did not lead to DNA ladders, and the proportion of apoptotic cells in the TUNEL assay was consistently < 3.5%

| | | | | MGH-U1/neo | | |
|---|---|---|---|---|---|---|
| **Drug** | **Dose** | **Duration** | **Interval to assay** | **DNA ladders** | **TUNEL**[a] | **Survival fraction**[b] |
| Doxorubicin | 0.9 µM | 1 h | 24 h | neg | 0.4% | 0.22 ± 0.06 |
| | 1.8 µM | 1 h | 24 h | neg | 0.2% | 0.045 ± 0.02 |
| Etoposide | 20 µM | 1 h | 24 h | neg | 0.2% | 0.124 ± 0.057 |
| | 40 µM | 1 h | 24 h | neg | 0.4% | 0.024 ± 0.015 |
| Cisplatin | 10 µM | 24 h | imm | neg | 0.3% | $3.0 \pm 0.5 \times 10^{-4}$ |
| | 20 µM | 24 h | imm | neg | 0.3% | $3.0 \pm 0.3 \times 10^{-4}$ |
| Ara C | 50 µM | 24 h | imm | neg | 5.6% | 0.72 ± 0.14 |
| | 100 µM | 24 h | imm | neg | 6.2% | 0.69 ± 0.11 |
| Gemcitabine | 50 µM | 24 h | imm | neg | 7.4% | 0.011 ± 0.005 |
| | 100 µM | 24 h | imm | neg | 7.4% | 0.011 ± 0.001 |

[a]Percent apoptotic cells by TUNEL assay; [b]± SEM; imm = immediate; neg = no ladders seen.

**Table 2** Summary of experiments to detect apoptotic markers and survival fraction (using a colony-forming assay) for CHO cells treated with a variety of anti-cancer drugs. Markers of apoptosis were not observed when CHO cells were treated with a variety of anti-cancer drugs for extended period of time at intervals from 0 to 6d (data not shown)

| | | | | CHO | | |
|---|---|---|---|---|---|---|
| **Drug** | **Dose** | **Duration** | **Interval to assay** | **DNA ladders** | **TUNEL** | **Survival fraction** |
| Doxorubicin | 0.18 µM | 18 h | imm | neg | NA | 0.12 ± 0.03 |
| | 0.45 µM | 18 h | imm | neg | NA | $1.0 \pm 0.1 \times 10^{-3}$ |
| | 0.9 µM | 1 h | 24 h | NA | 0.5% | $5.0 \pm 0.2 \times 10^{-4}$ |
| | 1.8 µM | 1 h | 24 h | NA | 3.6% | NA |
| Etoposide | 20 µM | 1 h | 24 h | NA | 0.7% | 0.34 ± 0.05 |
| | 40 µM | 1 h | 24 h | neg | 0.3% | 0.46 ± 0.13 |
| Cisplatin | 10 µM | 24 h | imm | NA | 0.2% | $5.0 \pm 0.6 \times 10^{-4}$ |
| | 20 µM | 24 h | imm | neg | 0.2% | 0 |
| Ara C | 50 µM | 24 h | imm | NA | 0.2% | 0.74 ± 0.04 |
| | 100 µM | 24 h | imm | NA | 0% | 0.08 ± 0.03 |
| Gemcitabine | 50 µM | 24 h | imm | neg | 0.2% | $3.0 \pm 0.3 \times 10^{-3}$ |
| | 100 µM | 24 h | imm | neg | 0.2% | $2.0 \pm 0.7 \times 10^{-3}$ |
| Overgrown cells | | | imm | pos | 57% | NA |

NA = Not available.

**Table 3** Summary of experiments to detect apoptotic markers for CTLL-2 cells treated with a variety of anti-cancer drugs

| | | | | CTLL-2 | |
|---|---|---|---|---|---|
| **Drug** | **Dose** | **Duration** | **Interval to assay** | **DNA Ladders** | **TUNEL** |
| Doxorubicin | 0.9 µM | 1 h | 24 h | neg | 2.1% |
| | 1.8 µM | 1 h | 24 h | neg | 1.1% |
| Etoposide | 20 µM | 1 h | 24 h | neg | 12% |
| | 40 µM | 1 h | 24 h | neg | 18% |
| Cisplatin | 10 µM | 24 h | imm | pos | 1.1% |
| | 20 µM | 24 h | imm | pos | 0.8% |
| Ara C | 50 µM | 24 h | imm | neg | 0.9% |
| | 100 µM | 24 h | imm | neg | 2% |
| Gemcitabine | 50 µM | 24 h | imm | pos | 0.6% |
| | 100 µM | 24 h | imm | pos | 6.9% |
| IL2 withdrawal | | 24 h | imm | pos | 38% |

© 2001 Cancer Research Campaign 

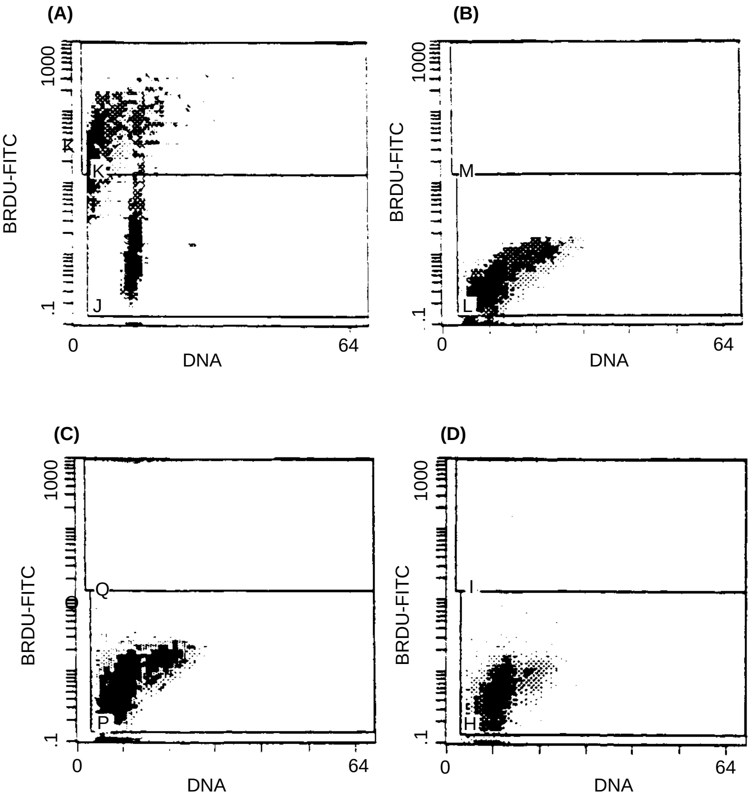

**Figure 4** Distribution of BrdU-FITC fluorescence versus DNA fluorescence with propidium iodide obtained by flow-cytometry after the TUNEL assay. In panel (**A**) (overgrown CHO cells) apoptotic cells have increased FITC fluorescence. In panels (**B**) (control CHO cells), (**C**) (CHO cells treated 24 h earlier with doxorubicin 1.85 μM × 1 h) and (**D**) (CHO cells treated 24 h earlier with etoposide, 40 μM × 1 h), FITC fluorescence did not increase

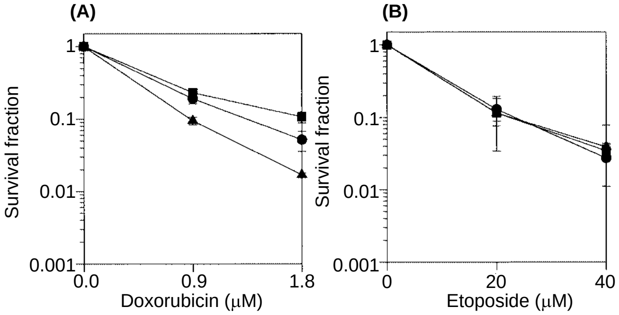

**Figure 5** Survival fraction (measured by a colony-forming assay) for MGH-U1/neo (●), MGH-U1/bax (▲) and MGH-U1/bcl-2 (■) cells following 1 hour treatments with (**A**) doxorubicin and (**B**) etoposide. Symbols indicate mean and standard error from 3 independent experiments influence sensitivity to these agents. For example, inhibition of the bcl-2 protein in tumour cells, or up-regulation of the bax protein or of caspases might be used to increase killing of tumour cells, and would be of therapeutic value if these manipulations could be undertaken selectively in tumour tissue. Conversely, up-regulation of *bcl-2* or of other genes that protect against apoptosis might be important causes of resistance to drugs or radiation. However, as illustrated conceptually in Figure 1, the observation of apoptotic markers after treatment with radiation and drugs is not strong evidence of their having a primary role in cell death. If the apoptotic process is initiated in cells that have undergone lethal and irreversible damage, markers of apoptosis may indicate only the process of cell lysis in cells that have already lost reproductive potential.

Most of the published evidence relating to the role of apoptosis following treatment with anti-cancer drugs or radiation is indirect. Many authors have simply recorded the presence of various markers of apoptosis and have assumed either explicitly or implicitly that there is a cause and effect relationship between their expression and loss of cell viability. Other studies have correlated the expression of pro-or anti-apoptotic genes in a variety of human cancers with their overall responsiveness to therapy: some have found a positive relationship (Kramer et al, 1996; Tai et al, 1998) whereas others have not (Pereira et al, 1997; Veronese et al, 1998). Even in studies which show a positive correlation, a cause and effect relationship has not been established.

In the present paper we have compared directly the loss of reproductive potential following treatment with anti-cancer agents with the appearance of apoptotic markers in CHO, MGH-U1 and CTLL-2 cells. Apoptosis is known to depend on p53 status, with immediate activation of apoptosis following DNA damage depending on the presence of wild-type p53. The MGH-U1 (and CTLL-2) cells are known to contain wild-type p53 whereas CHO cells are known to be mutant for p53 (Hu et al, 1999; Chang and Lai, 2000). However, all of these cells were quite sensitive to several anti-cancer drugs in

*© 2001 Cancer Research Campaign*

common use, yet apoptotic markers were apparent in at most a small fraction of the population even when clonogenic survival was in the range of $10^{-4}$ to $10^{-1}$. Also we were able to transfect and express at high levels the pro-apoptotic human gene *bax* and the anti-apoptotic human gene *bcl-2* in human MGH-U1 cells but found either no or minor effects on their clonogenic survival after treatment with etoposide and doxorubicin. These results are in accord with those of several other studies (Aldridge et al, 1995; Yin and Schimke, 1995; Lock and Strabinskiene, 1996; Kyprianou et al, 1997; Beale et al, 1998) although we were able to find two studies where changes in expression of genes that control apoptosis was able to modify clonogenic survival: for treatment of leukaemia cells with cytosine arabinoside (Hu et al, 1995) and for treatment of human breast cancer MCF-7 cells with radiation (Sakakura et al, 1996).

The role of apoptosis in mechanisms leading to loss of viability (in the important sense of loss of reproductive potential) following anti-cancer treatment is probably cell-line and drug-dependent. However, several studies, including the one presented here, suggest little or no role of apoptotic mechanisms in the killing of several types of cells by anti-cancer drugs beyond the trivial one of promoting lysis of cells that are already reproductively dead. The common assumption that the observation of apoptosis after treatment with anti-cancer agents implies its importance in determining therapeutic sensitivity or resistance may not be justified.

### ACKNOWLEDGEMENT

Supported by a research grant from the National Cancer Institute of Canada

### REFERENCES

Aldridge DR, Arends MJ and Radford IR (1995) Increasing the susceptibility of the rat 208F fibroblast cell line to radiation-induced apoptosis does not alter its clonogenic survival dose-response. *Br J Cancer* **71**: 571–577

Beale P, Rogers P, Hobbs S, Boxall F and Kelland L (1998) Transfection of bax into resistant human ovarian carcinoma cell lines confers no sensitisation to chemotherapeutic agents. *Br J Cancer* **78** (Suppl. 1): 30

Brezden CB, McClelland RA and Rauth AM (1994) Mechanism of the selective hypoxic cytotoxicity of 1-methyl-2-nitroimidazole. *Biochem Pharmacol* **48**: 361–370

Brown JM and Wouters BG (1999) Apoptosis, p53, and tumor cell sensitivity to anticancer agents. *Cancer Res* **59**: 1391–1399

Chang FL and Lai MD (2000) The relationship between p53 status and anticancer drugs-induced apoptosis in nine human bladder cancer cell lines. *Anticancer Res* **20**: 351–355

Hu T, Miller CM, Ridder GM and Aardema MJ (1999) Characterization of p53 in Chinese hamster cell lines CHO-K1, CHO-WBL, and CHL: implications for genotoxicity testing. *Mutat Res* **426**: 51–62

Hu ZB, Yang GS, Li M, Miyamoto N, Minden MD and McCulloch EA (1995) Mechanism of cytosine arabinoside toxicity to the blast cells of acute myeloblastic leukemia: involvement of free radicals. *Leukemia* **9**: 789–798

Kramer MH, Hermans J, Parker J, Krol AD, Kluin-Nelemans JC, Haak HL, van Groningen K, van Krieken JH, de Jong D and Kluin PM (1996) Clinical significance of bcl2 and p53 protein expression in diffuse large B-cell lymphoma: a population-based study. *J Clin Oncol* **14**: 2131–2138

Kyprianou N, King ED, Bradbury D and Rhee JG (1997) bcl-2 over-expression delays radiation-induced apoptosis without affecting the clonogenic survival of human prostate cancer cells. *Int J Cancer* **70**: 341–348

Lock RB and Strabinskiene L (1996) Dual modes of death induced by etoposide in human epithelial tumor cells allow Bcl-2 to inhibit apoptosis without affecting clonogenic survival. *Cancer Res* **56**: 4006–4012

Pereira H, Silva S, Juliao R, Gracia P and Perpetua F (1997) Prognostic markers for colorectal cancer: expression of p53 and BCL2. *World J Surg* **21**: 210–213

Roper PR and Drewinko B (1976) Comparison of in vitro methods to determine drug-induced cell lethality. *Cancer Res* **36**: 2182–2188

Sakakura C, Sweeney EA, Shirahama T, Igarashi Y, Hakomori S, Nakatani H, Tsujimoto H, Imanishi T, Ohgaki M, Ohyama T, Yamazaki J, Hagiwara A, Yamaguchi T, Sawai K and Takahashi T (1996) Overexpression of bax sensitizes human breast cancer MCF-7 cells to radiation-induced apoptosis. *Int J Cancer* **67**: 101–105

Smets LA (1994) Programmed cell death (apoptosis) and response to anti-cancer drugs. *Anti-cancer Drugs* **5**: 3–9

Tai YT, Lee S, Niloff E, Weisman C, Strobel T and Cannistra SA (1998) BAX protein expression and clinical outcome in epithelial ovarian cancer. *J Clin Oncol* **16**: 2583–2590

Veronese S, Mauri FA, Caffo O, Scaioli M, Aldovini D, Perrone G, Galligioni E, Doglioni C, Dalla Palma P and Barbareschi M (1998) Bax immunohistochemical expression in breast carcinoma: a study with long term follow-up. *Int J Cancer* **79**: 13–18

Yin DX and Schimke RT (1995) BCL-2 expression delays drug-induced apoptosis but does not increase clonogenic survival after drug treatment in HeLa cells. *Cancer Res* **55**: 4922–4928

© 2001 Cancer Research Campaign 
